# Faith and Science at the Hospitals

**Published:** 1892-06-11

**Authors:** 


### The Hospital

An Institutional Journal of

Science, Medicine, Nursing, and Philanthropy.

*For Hospitals, Asylums, Infirmaries, Dispensaries, Medical Practitioners, Students, Nurses, and the Charitable Public.*

Vol. XII.—No. 298.] [June 11, 1892.

### Faith and Science at the Hospitals.

One of the advantages of concrete and established institutions is that they give us a sense of fixity and permanence in a changeful and kaleidoscopic world. The activity of the young lions of science is so preternatural that a venerable Spencer threatens to be pushed on one side, and even the authority of a Darwin will not probably endure for more than another decade. But the science which is practical, like that of the treatment of the sick in hospitals, and not merely speculative, suffers nothing by the dethronement of great scientific kings, and the upheavals which destroy their dynasties. The practical roots itself more and more firmly, like the spreading oak, under the stormy tossing of the winds of fitful theory.

The most conservative among us cannot but feel that we are living in an age of movement, and the movement is forward. Many ancient things have gone by the board, and many more will go. But every man has a natural longing for certainty. Endless change is tiresome; it is paralysing to all that is strong and good. Why seek truth if we can never find it? Why weary the brain in the search after knowledge if the theory we have mastered so painfully to day is to be set aside by a new and equally uncertain theory to-morrow? The practical is not open to these objections. When a man with a broken leg has had his limb properly "set," and has stayed his five or six weeks in the hospital ward, he is restored to activity and usefulness; and the very cleverest of young speculative thinkers will hesitate before he throws doubt on the validity of the cure.

The practical renders the very highest service to speculative science. It not only plays the part of judge and jury, but of judge and jury that are infallible. Hospitals are of necessity in the very forefront of ascertained science. The inconsequence, not to say whimsicality, of even scientific minds is sometimes so great that practical tests are absolutely imperative, if speculative science is to preserve its sanity. Men, even clever men, will think the maddest thoughts; and some of them will not only think but publish their impossible extravagances. This has been in times past emphatically the case with doctors and medicine. But now we have the hospitals as sure testing-places; and it is astonishing how, like the purifying fire, they "try every man's work of what sort it is."

The religious world, which has supported hospitals out of pure disinterestedness, little knows what a barrier it has thus raised up against the too aggressive activity of a purely secular and inexperienced science. Religion has often essayed to fight science with the weapons of criticism and logic; and she has wasted a great deal of her strength on enemies which were nothing more than shadows. If she could have pointed to some scientific testing ground, and demanded that science should test her theories there before assuming them to be permanently true and sound, how many anxious tremors and battles of an exhausting kind she would have been spared. It is a thing well worth thinking of, that in the admittedly utilitarian and virtuous work of healing the sick, science allows religion and philanthropy to take the lead, and is content to act as their handmaids. Science is thus compelled to stand face to face with religion in its solid and concrete form, and in that form the very profoundest and most critical of scientific men have not a word to say against religion. This, too, is worth thinking of; if the material body of religion, as thus seen in its philanthropic and virtuous work, is so entirely good, is there not more than a presumption that the essential spirit is also and equally good?

The meeting of religion and science on the common ground of hospital work is of the greatest advantage to both. Religion needs a testing ground as well as science. With the average man it tends to run to theory, to exercise the speculative faculty, and to sever itself from practice. An institution like the hospital system gives the religious man an opportunity of using his intelligence and his judgment in the carrying out of his principles. Religion is intellectual in the intellectual man. Now to the intellectual man there are things greater and things smaller, things more important and things less important in every department of human life. The intellectual man cannot help weighing and measuring; he never mistakes a marble for an orange, nor an orange for a cannon ball. But the unintellectual religious man constantly makes mistakes of this order. His religion is an unmeasured and undefined sentiment, into which he puts no brains. The hospital system gives him an opportunity of putting some intellect into his concrete religion. He has, possibly, in past times regarded that system as a philanthropic marble or orange, whereas its real magnitude is that of the very largest of cannon balls, and the support which he ought to give to it is to be measured accordingly.

For a new movement enthusiasm and an irresistible impulse are the things desired; for an established institution a critical judgment and a clear vision are the faculties more demanded. The hospitals invite a critical judgment; they have everything to gain by a clear vision and full knowledge. We have published arrays of facts in this journal before; we elsewhere publish similar facts on the present occasion, and these are the things to be studied. It seems to us that the simplicity and earnestness of the national religion may be measured by the amount of interest and support which it gives to such institutions as hospitals. There is nothing in them to arouse ecclesiastical passions; there is everything in them to awaken intelligence, sympathy, and duty. For our part we have been under the necessity of studying every department of hospital work for a considerable number of years, and as a result the conviction has gradually grown up in the mind that there is no work more truly divine in itself, and more entirely satisfying to that high intelligence which is purified from worldly passions and ambitions than the work of hospitals.

There is something about simple benevolence and good deeds that represses strife. The word said may be denied; the deed done cannot. To feed the really hungry is a thing which nobody blames; to heal the really sick commands still more approval. It is one thing which always shines as a most conspicuous merit in hospitals, that they do actually accomplish the work they take in hand; and this, notwithstanding that the work itself is so great, various, and difficult. The men and women who have the work in hand are deeply in earnest. In this respect they set an example to the whole world, even to the world of religion. Their work does not consist in words and beautiful emotions, but in unremitting watchfulness and effective deeds. Medicine, in truth, is a real religion. The earnest physician has a very great deal in common with the earnest priest and the faithful teacher. It is a striking fact that, notwithstanding the frequent antagonism between men of science and men of faith, the often opposed camps should have always agreed to sheath their weapons at the bedside of suffering and of pain.

A great effort has been initiated by the hospitals during the present year whereby they hope to bring their resources abreast of the necessities of the times. In this they are emulating the example of all living Churches. Religious and philanthropic men have hitherto led the van of care for the helpless and the sick. It would be a pity amounting to a portent if they were now to fall into the rear. It would show a diminishing energy in practical religion and a growing secularisation of the people. But the people cannot afford to be less religious; they need to be more religious. A practical nation like our own not only best understands but best appreciates a practical religion. We believe that if the churches would make an unparalleled effort and give to the forward movement initiated by the hospitals such a mighty impulse as they have abundant power to give, not only would the hospitals find themselves rising to the summit of a wave of unparalleled prosperity, but the churches themselves would be more blessed than the hospitals by the quickening and enduring impulse of their own effort.

